# Risk Factors for Preeclampsia in Women from Colombia: A Case-Control Study

**DOI:** 10.1371/journal.pone.0041622

**Published:** 2012-07-23

**Authors:** Laura M. Reyes, Ronald G. García, Silvia L. Ruiz, Paul A. Camacho, Maria B. Ospina, Gustavo Aroca, Jose L. Accini, Patricio López-Jaramillo

**Affiliations:** 1 Department of Research, Fundación Cardiovascular de Colombia, Floridablanca, Santander, Colombia; 2 School of Public Health, University of Alberta, Edmonton, Alberta, Canada; 3 Clínica de la Costa, Barranquilla, Atlántico, Colombia; 4 Hospital Universitario de Barranquilla, Barranquilla, Atlántico, Colombia; 5 Department of Research, Faculty of Medicine, Universidad de Santander (UDES), Bucaramanga, Santander, Colombia; 6 Department of Research, Fundación Oftalmológica de Santander-Clínica Carlos Ardila Lulle (FOSCAL), Floridablanca, Santander, Colombia; Tehran University of Medical Sciences, Islamic Republic of Iran

## Abstract

**Background:**

Preeclampsia (PE) is a multi-causal disease characterized by the development of hypertension and proteinuria in the second half of pregnancy. Multiple risk factors have been associated with the development of PE. Moreover, it is known that these risk factors vary between populations from developed and developing countries. The aim of this study is to identify which risk factors are associated with the development of preeclampsia (PE) among Colombian women.

**Methods:**

A multi-centre case-control study was conducted between September 2006 and July 2009 in six Colombian cities. Cases included women with PE (n = 201); controls were aged-matched pregnant women (n = 201) without cardiovascular or endocrine diseases for a case-control ratio of 1∶1. A complete medical chart, physical examination and biochemical analysis were completed before delivery. Multivariable logistic regression was used to calculate odds ratios (OR) and 95% confidence intervals (CI) of potential risk factors associated with PE.

**Results:**

The presence of factors present in the metabolic syndrome cluster such as body mass index >31 Kg/m2 (OR = 2.18; 1.14–4.14 95% CI), high-density lipoprotein <1.24 mmol/L (OR = 2.42; 1.53–3.84 95% CI), triglycerides >3.24 mmol/L (OR = 1.60; 1.04–2.48 95% CI) and glycemia >4.9 mmol/L (OR = 2.66; 1.47–4.81 95%CI) as well as being primigravidae (OR = 1.71; 1.07–2.73 95% CI) were associated with the development of PE, after adjusting for other variables.

**Conclusion:**

Factors present in the cluster of metabolic syndrome and primigravidity were associated with a greater risk of PE among Colombian women. Understanding the role of this cluster of risk factors in the development of PE is of crucial importance to prevent PE and remains to be determined.

## Introduction

Preeclampsia (PE) is a pregnancy-related hypertensive disorder occurring after 20 weeks of gestation. It constitutes a significant public health problem in both industrialized and developing countries, contributing to maternal and perinatal morbidity and mortality worldwide. PE affects between five to ten percent of pregnancies [1], [2]. The impact of the condition, however, is thought to be more severe in developing countries. The prevalence of PE in developing countries has been estimated between 1.8% and 16.7% [3] with at least 16% of maternal deaths resulting from PE [4].

In spite of the detrimental effects of PE on maternal and neonatal outcomes, understanding the etiology and mechanisms of the disease remains a major challenge. Evidence compiled from controlled studies has shown that potential risk factors for PE include a family or personal history of PE, the presence of antiphospholipid antibodies, pre-existing diabetes, multiple pregnancies, nulliparity, hypertension or raised blood pressure at antenatal visits, increased body mass index (BMI) before or during pregnancy and advanced maternal age (greater than 40 years of age) [5], [6]. Similarly, there is evidence that the risk of PE increases with an interval of 10 years or more between pregnancies, autoimmune disease, renal disease, and chronic hypertension [5].

The relative importance of risk factors leading to the occurrence of PE depends on the level of socioeconomic development reached by a given society [7]. In developing countries, the fast socioeconomic transition and the adoption of a Westernized lifestyle have increased the exposure to a high-saturated fat diet, sedentary lifestyles, smoking and psychosocial stress. These factors result in the development of obesity, hypertension and changes in carbohydrate metabolism that may be associated with the epidemic of cardio-metabolic diseases in these countries from an early age [7]–[9]. The factors mentioned above put women of childbearing age at a higher risk for the development of a spectrum of multiple diseases from anovulation [10] to gestational diabetes [11] and PE [12]. Little research has been conducted to specifically identify risk factors for the occurrence of PE among pregnant women in developing countries. Understanding which factors can be prevented or controlled within a population under rapid social, economic and structural changes is of crucial importance from a public health perspective [13]. The aim of this study was to identify which risk factors are associated with the development of PE among Colombian women.

## Methods

### Ethics statement

The Fundación Cardiovascular de Colombia Institutional Ethics Review Board approved the study protocol and written informed consent was obtained from all study participants before interviews, clinical assessments and biochemical analysis were conducted.

### Study population

A multi-centre case–control study was conducted among women who delivered singleton infants in seven hospitals from six Colombian cities (Bucaramanga, Medellín, Envigado, Cúcuta, Barranquilla and Santa Marta) between September 2006 and July 2009. Sub-analyses from this study have been published elsewhere [14], [15]. Briefly, cases were identified by monitoring of all admissions to obstetric services of participating centres. A total of 201 women with a confirmed diagnosis of PE [blood pressure of ≥140/90 mmHg after the 20th week of gestation in addition to the presence of proteinuria (urinary protein ≥300 mg in a 24-hour period or ≥1+ on dipstick in at least two random urine samples taken at least 4–6 hours apart)] [16], [17] were recruited.

The control group was a consecutive convenience sample of healthy pregnant women (n = 201) without evidence of PE or any cardiovascular or endocrine disease before or during pregnancy. They were selected and recruited from the same hospital of delivery as PE cases. Cases and controls were frequency matched by age (± three years), centre and ethnicity. The following conditions were considered as exclusion criteria for both groups: history of chronic hypertension or cardiovascular diseases, endocrine diseases, autoimmune diseases, renal diseases, mental illness, human immunodeficiency virus (HIV) and cancer.

### Clinical assessment

A complete chart including obstetric, family and socio-economical history was recorded using a standardized format prior to delivery. The investigator classified each participant's ethnicity in order to assess whether the factors associated to the development of PE were related to their ethnicity. Psychological stress was assessed with two questions related to the presence of permanent stress at work and at home in the previous 12 months. Standard measurements were performed in duplicate by the same examiner on each woman. All physical measurements were taken before the delivery, with women wearing light clothes and without shoes.

### Biochemical determinations

Blood samples from the antecubital vein were withdrawn in fasting conditions (6 hours) before delivery. Plasma was separated by centrifugation at 3000 rpm for 15 minutes and then frozen at −70°C until analysis. An aliquot of whole blood was collected to determine hematocrit, hemoglobin, leukocyte count, and glucose concentration (Baker System 9120 AX, Biochem Immunosystem, Allentown, PA). The lipid profile (high density lipoprotein [HDL], low density lipoprotein [LDL], very low density lipoprotein [VLDL], triglycerides [TG]) was determined by enzyme colorimetric test (Biosystems BTS-303 Photometric, Barcelona, Spain). C reactive protein (CRP) was determined by chemiluminescent immunometric assay (IMMULITE, Automatic Analyzer, Diagnostic Products Corporation, L.A., USA). Standard microscopic examinations were performed after centrifugation of all urine samples. Urocultures were performed to disregard suspected urinary tract infections.

### Statistical analysis

Descriptive analyses were reported as proportions and percentages for categorical data, and means with standard deviations (SD) or median and interquartile range for continuous data. The Shapiro-Wilk test was used to assess the normality of continuous data. Univariate analysis of continuous data was conducted using two-sample *t* tests or Mann-Whitney test (for non-normally distributed data). Categorical variables were analyzed using *x^2^* test or Fisher's exact test. Statistical significance was defined as p<0.05. Multivariable logistic regression procedures were employed to calculate odd ratios (OR) of potential risk factors for PE.

This research is part of an international, multi-centre case-control study aimed at evaluating the presence of risk factors associated with the development of PE among pregnant women in developing countries. An overall sample size calculation was made for the original 20 participating countries. Assuming an exposure rate of 4% per risk factor in the control group, a total sample of 6,957 pregnant women (348 per country, approximately) would provide 80% power at the α = 0.05 level to detect a clinically relevant OR of 1.4 for each factor. Adjustments for a missing data rate of 10% were made for a total sample size of 7,709 study participants (385 per country). To guarantee enough statistical power, it was decided to include a total of at least 4000 women with PE and 4000 healthy pregnant women (i.e., 200 cases and 200 controls per country), for a case-control ratio of 1∶1.

A forward logistic regression model was conducted to explore whether familial history of cardiovascular diseases in first degree, familial history of PE or eclampsia, being primigravidae, permanent work or home-related stress, condom use, vitamin or folic acid supplementation during pregnancy, leukocyte count >10×10^9^/L, CRP>28.57 nmol/L and the multiple components of the MS cluster (glycemia, TG, HDL, BMI) were factors associated with the development of PE. Cut-off points proposed by Dane et al. [18], to determine metabolic syndrome (MS) in pregnant women were used to dichotomize the following variables for the logistic regression model: glycemia >4.9 mmol/L, TG>3.24 mmol/L, HDL <1.24 mmol/L, body mass index (BMI)>31 kg/m^2^.

Variables were tested for collinearity before entering the model. A p value less than 0.2 was chosen in the univariate analysis as a criteria to enter the multivariate model. The Hosmer-Lemeshow chi-square test (p = 0.728) was used to assess the goodness of fit of the model. Data imputation was carried out for missing values for the variables BMI and glycemia. For BMI, data imputation was based on the mean age and mean gestational age of all study participants; data imputation for glycemia was based on the mean glycemia and mean BMI. Confidence intervals at the 95% level (95% CI) were reported for the adjusted ORs. All analyses were completed using STATA software, version 10.0 (Stata Corp., 2007, College Station, TX).

## Results

Socio-demographic, behavioral, clinical and biochemical characteristics of women with PE and healthy pregnant women are presented in Table 1. Overall, there were no statistically significant differences between cases and controls in the mean age, ethnicity, and household income.

**Table 1:** Socio-demographic, clinical and biochemical characteristics of women with preeclampsia and healthy pregnant women.

	Women with preeclampsia	Healthy pregnant controls	p value
	n = 201	n = 201	
**Socio-demographic characteristics**			
Age (years)	26.5±7.2	26.7±7.2	0.728
Ethnicity (%)			0.918
Mixed	80.8	81.4	
White	12.4	13.5	
Black from African caribbean ancestry	2.6	1.6	
Aboriginal	4.2	3.6	
Education (%)			0.257
None	0	1.5	
High school	67.5	64.8	
University	32.5	33.7	
Household Income (median [interquartile range])	363 [475.2–610.8]	414.9 [556.5–722.9]	0.07
**Clinical characteristics**			
Primigravidae proportion (%)	36.9	25.9	**0.019**
Gestational age (weeks)	34.1±4.2	37.7±3.5	**<0.001**
SBP (mm Hg)	151±14	112±6	**<0.001**
DBP (mm Hg)	95±9	68±6	**<0.001**
Heart rate (bpm)	84±13	79±8	**<0.001**
Weight (Kg)	71.1±13.4	68.1±10.8	**0.021**
Height (centimeters)	158.9±6.5	158.8±13.3	0.131
BMI (Kg/m^2^)	27.9±5.0	26.7±3.8	**0.028**
**Behavioural factors** (**%**)			
Use condoms as contraceptive with the father of the unborn baby	85.1	71.8	**0.006**
Calcium supplementation during pregnancy	49.2	52.2	0.559
Iron supplementation during pregnancy	49.1	52.8	0.615
Vitamins supplementation during pregnancy	43.6	55.1	**0.025**
Folic acid supplementation during pregnancy	47.6	60.3	0.073
Permanent work-related stress	21.51	19.8	0.155
Permanent home-related stress	8.1	2.5	**0.045**
Alcohol consumption during pregnancy	18.9	15.7	0.410
Smoking during pregnancy	2.1	3.5	0.274
**Family history** (**%**)			
Mother with PE history	5.5	3.2	0.291
Sisters with PE history	9.1	4.4	0.117
Mother with Eclampsia history	1.1	0.5	0.490
Sisters with Eclampsia history	0.6	0.7	0.717
Mother with HELLP syndrome history	0	0.5	0.508
Sisters with HELLP syndrome history	0	0.7	0.467
History of hypertension among first-degree family members	48.2	29	**<0.001**
History of dyslipidemia among first-degree family members	29.5	18.6	**0.019**
History of diabetes among first-degree family members	25	14.8	**0.017**
History of coronary heart disease among first-degree family members	22.8	11.9	**0.007**
History of stroke among first-degree family members	14.3	5.1	**0.004**
**Biochemical characteristics**			
Hemoglobin (g/dL) (median [interquartile range])	11.9 [10.8–13.0]	12.1 [11.0–12.9]	0.405
Hematocrit (%)(median [interquartile range])	35.1 [32.1–38.5]	36.15 [33.2–38.7]	0.076
Platelets (×10^9^/L) (median [interquartile range])	207.0 [169.5–270.5]	243.0 [199.0–287.0]	**<0.001**
Leukocyte count (×10^9^/L) (median [interquartile range])	10.7 [8.8–13.4]	9.8 [8.0–11.6]	**<0.001**
Neutrophils (%)(median [interquartile range])	74.8 [69–82]	73 [69–78]	0.106
Lymphocytes (%)(median [interquartile range])	20 [14]–[26]	21 [16]–[25]	0.307
Total Cholesterol (mmol/L) (median [interquartile range])	6.3 [5.1–7.3]	6.7 [5.76–7.71]	**0.001**
HDL (mmol/L) (median [interquartile range])	1.3 [1.0–1.6]	1.4 [1.2–1.7]	**0.001**
VLDL (mmol/L) (median [interquartile range])	1.5 [1.1–1.9]	1.3 [1.1–1.7]	**0.017**
LDL (mmol/L) (median [interquartile range])	3.3 [2.4–4.1]	3.8 [2.7–4.6]	**<0.001**
Triglycerides (mmol/L) (median [interquartile range])	3.3 [2.5–4.2]	3.0 [2.4–3.8]	**0.017**
Glycemia (mmol/L) (median [interquartile range])	4.3 [3.6–5.0]	4.0 [3.5–4.5]	**<0.001**
C reactive protein (nmol/L) (median [interquartile range])	80.9 [40.9–216.1]	59.0 [31.4–119.0]	**<0.001**
Positive uroculture (%)	44	4.55	**0.002**

Results expressed as mean ± standard deviation.

BMI: body mass index, DBP: diastolic blood pressure; HDL: high-density lipoprotein; HELLP: hemolysis, elevated liver enzymes, low platelet count; LDL: low-density lipoprotein; PE: preeclampsia; SBP: systolic blood pressure; VLDL: very-low-density lipoprotein.

Regarding their clinical characteristics, the proportion of primigravidae (36.8% versus 25.8; p = 0.019), and mean systolic blood pressure (151 versus 112 mm Hg; p<0.001), diastolic blood pressure (95 versus 68 mm Hg; p<0.001), heart rate (84 versus 79 bpm; p<0.001) weight (71.1 versus 68 kg) and BMI (27.9 versus 26.6 kg/m^2^) were significantly greater among PE women. Alternatively, the average gestational age was statistically significantly higher among healthy pregnant women compared to women with PE (34 versus 37.6 weeks; p<0.001).

Compared to healthy pregnant women, women with PE were more likely to use condoms as a contraceptive method with the father of the unborn baby. Of all study participants, 96.4% reported attending prenatal care, with a mean of 6 visits per woman. No significant differences were found in the attendance at prenatal clinics. There were no significant differences in the proportion of oral supplementation with calcium, iron and folic acid prescribed during prenatal care visits. Compared to healthy pregnant women, women with PE were less likely to have received vitamin supplementation during prenatal care (43.5% versus 55.1%; p = 0.025). Alcohol consumption and smoking were not statistically different between the groups.

There were no statistical differences between cases and controls in the proportion of work-related stress. Nonetheless, women with PE had a higher rate of home-related stress during the previous 12 months compared to healthy pregnant women (8.08% versus 2.54%; p = 0.045). The proportion of hypertension, dyslipidemia, diabetes, coronary heart disease and stroke among first-degree family members was significantly higher among cases. There were no significant differences between the groups regarding family history of PE, eclampsia or HELLP (hemolysis, elevated liver enzymes) syndrome.

Women with PE had a significantly lower platelet count and higher white blood cell count compared to controls. There were significant differences between CRP levels, which were higher among the cases. Only 13.7% of all participants had a positive urine analysis. Women with PE had a higher proportion of positive urine cultures compared to healthy pregnant women (44 versus 4.5%; p = 0.002). The most commonly isolated germ was Escherichia coli, followed by Enterobacter and Klebsiella.

Women with PE had higher fasting blood glucose values compared to healthy pregnant women (p<0.0001). With regard to lipid profile, it was found that women with PE had lower HDL cholesterol levels and higher triglyceride levels compared to healthy pregnant women.

Results from the logistic regression analysis are summarized in Table 2. The presence of multiple components of the MS cluster (glucose >4.9 mmol/L [OR: 2.66; 95% CI: 1.47–4.81], TG>3.24 mmol/L [OR: 1.60; 95% CI: 1.04, 2.48], HDL <1.24 mmol/L [OR: 2.42; 95% CI: 1.53–3.84], BMI>31 kg/m^2^ [OR: 2.18; 95% CI: 1.14–4.14]) and being primigravidae [OR: 1.71; 95% CI: 1.07–2.73] remained as factors associated with the development of PE after adjusting for familial history of cardiovascular diseases in first degree; familial history of PE or eclampsia, vitamin supplementation during pregnancy, condom use, leukocyte count, CRP and home related-stress.

**Table 2:** Multivariate analysis of the risk factors associated with the development of preeclampsia.

	Unadjusted odds ratio	95% CI	Adjusted odds ratios	95% CI
Primigravidae proportion	1.67	1.06–2.63	1.71	1.07–2.73
Permanent work-related stress	0.71	0.28–1.82	.	.
Permanent home-related stress	3.12	0.97–11.76	.	.
Use condoms as contraceptive with the father of the unborn baby	2.24	1.2–4.24	.	.
Vitamins supplementation during pregnancy	0.62	0.41–0.95	.	.
Folic acid supplementation during pregnancy	0.59	0.32–1.09	.	.
Sisters with PE history	2.01	0.68–6.63	.	.
History of hypertension among first-degree family members	2.27	1.42–3.65	.	.
History of dyslipidemia among first-degree family members	1.82	1.06–3.12	.	.
History of diabetes among first-degree family members	1.97	1.08–3.64	.	.
History of coronary heart disease among first-degree family members	2.19	1.18–4.12	.	.
History of stroke among first-degree family members	3.09	1.33–7.78	.	.
BMI >31 Kg/m^2^	2.90	1.52–5.70	2.18	1.14–4.14
Leukocyte count >10×10^9^/L	1.74	1.14–2.67	.	.
HDL <1.24 mmol/L	2.29	1.46–3.61	2.42	1.53–3.84
LDL >2.59 mmol/L	0.56	0.56–0.91	.	.
Triglycerides >3.24 mmol/L	1.65	1.09–2.51	1.60	1.04–2.48
Glycemia >4.9 mmol/L	2.80	1.57–5.11	2.66	1.47–4.81
C reactive protein >28.57 nmol/L	1.31	0.76–2.30	.	.

BMI: body mass index; HDL: high-density lipoprotein; LDL: low-density lipoprotein; PE: preeclampsia. Reference group: Healthy pregnant controls. An OR greater than 1 indicates a greater likelihood of having the factor than the reference group while an OR under 1 indicates a lesser likelihood of the factor than the reference group.

## Discussion

Results of this case-control study of a large sample of pregnant women from a developing country showed that the presence of the multiple components of the MS cluster and being primigravidae are factors that are significantly associated with the development of PE.

PE has a large impact on public health in developing countries. Variations in the prevalence of PE among world regions have been attributed to low adherence to antenatal care or women that tend to show up late in pregnancy for antenatal care as a result of restricted access to maternity services, poor transportation systems and certain cultural practices [19]. Studies conducted in pregnant Colombian women, however, have demonstrated that chronic sub-clinical inflammation [20] and metabolic alterations [21] affect vascular endothelial function and increase the risk of PE. These findings are also supported by other studies in which pregnant women (<30 weeks) who subsequently developed PE, had higher CRP concentrations [22] when compared to healthy pregnant controls. Moreover, women who developed PE presented a higher degree of insulin resistance prior to the onset of clinical manifestations of the disease [21]. Furthermore, previous findings regarding the nutritional status of women from the present study [14] demonstrated that there was an association between increased carbohydrate and sodium consumption and the development of PE in this population. Hence, according to these results and results from the present study, adopting a Westernized lifestyle in developing countries has an enormous impact on maternal health that may be comparable or even higher than that of having restricted access to health services.

The pandemic of obesity has reached considerable proportions in developing countries, where the proportion of people who are overweight is as high as 40% [23]. There is evidence that being overweight leads to insulin resistance [24] and increases both inflammatory markers [25], [26] and the risk of developing cardiovascular diseases [27]–[29].

The association between obesity, lipid profile alterations, carbohydrate intolerance and PE development is well established. Driul et al. [30] conducted a retrospective cohort study of 916 consecutive singleton gestations to evaluate the association between pre-pregnancy BMI and adverse maternal and neonatal outcomes. The study demonstrated that obese women were five times more likely to develop PE. The excessive weight gain during pregnancy in women with normal pre-pregnancy BMI has been associated with increased rates of gestational hypertension [31]. Moreover, it has been determined that carbohydrate intolerance in pregnant women is associated with an increased risk of developing PE [32]–[34]. Along with obesity and carbohydrate intolerance, hypertriglyceridemia [35] and low HDL [36] have been associated with PE development. There is also a growing interest in studying the association between MS and PE because some of the risk factors associated with the development of MS, such as insulin resistance and dyslipidemia, are considered physiological adaptations of pregnancy [37], [38]. Dane et al. [18] evaluated the presence of MS in 41 women who developed pregnancy-induced hypertension compared to 97 healthy pregnant women and designed a metabolic scoring system to analyze the risk of hypertensive disorders of pregnancy. The cut-off values for metabolic scores were created using standard deviations in the normotensive group. The authors found that the presence of multiple components of MS is a risk factor for the development of pregnancy-induced hypertension. The results of the present study clearly demonstrate that the presence of the cluster of factors that comprise MS is an important risk factor associated with the development of PE after adjusting for other clinical and biochemical factors.

Regarding familial cardiovascular risk, previous studies have demonstrated that pre-existing cardiovascular risk factors, and particularly hypertension, increases a woman's susceptibility to developing hypertension in pregnancy [39], [40]. Bivariate analyses in our study revealed a significant association between a family history of cardiovascular diseases and the development of PE. After adjusting for other variables, however, results were not significant.

The importance of the results from the present study is that they suggest that most of the risk factors associated with PE are modifiable; they can be prevented, thus reducing the burden of PE in developing countries. To reach this goal, the health system must guarantee adequate nutrition and promote healthy lifestyles in pregnant women [41]. In addition, prenatal care must emphasize the control of factors responsible for the MS cluster.

The impact of controlling MS during pregnancy to reduce both the incidence of PE and the risk of developing cardio-metabolic diseases in the future remains to be determined. A recent meta-analysis by Bellami et al. [42] however, demonstrated that the risk of hypertension, ischemic heart disease, stroke and venous thromboembolism in women increases after a PE episode. The authors also found an increase in overall mortality after PE. These results, together with ours, suggest that the control of the multiple components of the MS cluster during pregnancy can have a positive effect for both the prevention of PE and the prevention of cardio-vascular diseases.

The effect that PE has on fetal programming and the risk of developing cardiovascular diseases later in life in children born following a pregnancy complicated by PE remains to be established. It has been proposed, however, that PE may have an important role in the epidemic of cardiovascular diseases and type-2 diabetes experienced in developing countries [43].

Recall bias, missing data and imputation of data were potential limitations in our study. Data imputation was conducted according to previously validated methods [44]. It is unlikely that data imputation overestimated our study results as the number of data that was imputed in each of the two variables with missing data was less than 15%.

### Conclusion

The presence of multiple components of the MS cluster and being primigravidae are risk factors associated with the development of PE among pregnant women from a developing country. This finding can help to guide maternal health policies aimed at preventing the development of a disease with a considerable economic and social impact such as PE.

PE prevention interventions should start before pregnancy and target women of childbearing age by reinforcing the adoption of healthy life style practices (i.e., dietary changes and exercise). Timely prenatal care during pregnancy will allow for early detection and treatment of unfavourable nutritional practices that lead to excessive weight gain and biochemical alterations of the lipid profile and carbohydrates.
